# Duration of acute kidney injury in critically ill patients

**DOI:** 10.1186/s13613-018-0374-x

**Published:** 2018-02-23

**Authors:** Christine K. Federspiel, Theis S. Itenov, Kala Mehta, Raymond K. Hsu, Morten H. Bestle, Kathleen D. Liu

**Affiliations:** 1 Division of Nephrology, Department of Medicine, University of California, San Francisco, Box 0532, San Francisco, CA 94143-0532 USA; 2 Department of Anesthesiology, Nordsjællands Hospital, University of Copenhagen, Copenhagen, Denmark; 3 Department of Epidemiology and Biostatistics, University of California, San Francisco, San Francisco, USA; 4 Division of Nephrology, Department of Medicine, University of California, San Francisco, San Francisco, USA; 5 Divisions of Nephrology and Critical Care Medicine, Departments of Medicine and Anesthesia, University of California, San Francisco, Box 0532, San Francisco, CA 94143-0532 USA

**Keywords:** Acute kidney injury, Intensive care, Acute respiratory distress syndrome, Sepsis

## Abstract

**Background:**

Duration of acute kidney injury (AKI) has been recognized a risk factor for adverse outcomes following AKI. We sought to examine the relationship of AKI duration and recurrent AKI with short-term outcomes in critically ill patients who were mechanically ventilated and met criteria for the acute respiratory distress syndrome.

**Methods:**

Participants in the NHLBI ARDS Network SAILS multicenter trial who developed AKI were included in this analysis and divided into groups based on AKI duration. Differences in outcomes were evaluated using *t* test and Chi-square test. Competing risks regression and Cox regression were used to evaluate factors associated with resolving AKI and recurrent AKI.

**Results:**

In total, 238 patients were included in the study. Seventy-seven patients had short duration AKI (1–2 days), 47 medium duration AKI (3–7 days), 87 persistent AKI (> 7 days) and 38 died during their AKI episode. Persistent AKI was associated with worse outcomes including increased ICU length of stay, time on the ventilator and days with cardiovascular failure. We found no clinical differences between patients with short and medium duration AKI, even when accounting for AKI severity and recurrent AKI. Patients with resolving AKI were less likely to have oliguria or moderate/severe ARDS on the day AKI criteria were met. Recurrent AKI was associated with poorer clinical outcomes. No baseline clinical factors were found to predict development of recurrent AKI.

**Conclusions:**

In critically ill patients with sepsis-associated ARDS and AKI, the impact of short and medium duration AKI on clinical outcomes was modest. Persistent and recurrent AKI were both associated with worse clinical outcomes, emphasizing the importance of identifying these patients, who may benefit from novel interventions.

**Electronic supplementary material:**

The online version of this article (10.1186/s13613-018-0374-x) contains supplementary material, which is available to authorized users.

## Background

Acute kidney injury (AKI) is a common illness in critically ill patients that is associated with increased morbidity and mortality [1–3]. The severity of AKI is typically graded by the magnitude of the serum creatinine rise and/or fall in urine output, and more severe AKI is associated with poorer outcomes [4]. Recently, the duration of AKI has been recognized as an important risk factor for adverse outcomes, where short duration AKI (also called rapid reversal or transient AKI), typically defined as less than 48–72 h, has been associated with a lower risk of chronic kidney disease (CKD), end-stage renal disease (ESRD) and mortality compared to AKI of longer duration [4–7]. However, a recent ICU-based study demonstrated that although persistent AKI was associated with an increased risk of in-hospital death, this relationship was attenuated after accounting for AKI severity [8].

The main aim of the current study was to further examine the relationship of AKI duration with clinical outcomes in a cohort of critically ill patients, accounting for AKI severity as well as for subsequent recurrent AKI. Given the lack of pharmacologic therapies for AKI and the urgent need to identify relevant patient populations for clinical trials, we sought to identify factors associated with resolving AKI as well as risk factors for development of recurrent AKI. For this analysis, we focused on patients with sepsis-associated acute respiratory distress syndrome (ARDS) who were enrolled in a clinical trial of rosuvastatin for ARDS [9].

## Methods

### Patient population

The Statins for Acutely Injured Lungs from Sepsis (SAILS) study was a randomized clinical trial conducted from March 2010 to September 2013 at 44 hospitals in the USA as part of the National Heart, Lung, and Blood Institute (NHLBI) ARDS Network (ClinicalTrials.gov NCT00979121) [9]. The trial tested the potential benefit of rosuvastatin on mortality in patients with sepsis-associated ARDS. Specific inclusion and exclusion criteria are described elsewhere [9]. Ventilator management and weaning along with fluid management after shock resolution were protocolized; specifically, ventilator management included a lung protective, low tidal volume ventilation strategy [9]. The trial was stopped early for futility; in the final analysis, there was no relationship of rosuvastatin therapy with mortality. For the SAILS study, the IRB at each site approved the study. The current study used de-identified data from the trial and was therefore exempt from IRB review.

Patients with known ESRD or who were on dialysis for AKI at study enrollment were excluded. Daily serum creatinine measurements were available for days 1–14 after study enrollment along with the maximum serum creatinine value from days 14 to 28 and all measurements from the 48 h prior to enrollment. AKI was defined according to the KDIGO serum creatinine criteria [10]. Baseline serum creatinine was defined as the lowest serum creatinine within the 48 h prior to randomization. AKI was defined as either a ≥ 1.5 times increase from baseline, or ≥ 0.3 mg/dL increase during a 48-h rolling window [10]. We included all study participants who developed AKI during the first 5 days of study enrollment, to allow for sufficient follow-up time to ascertain AKI duration.

### Duration of AKI

AKI duration was defined as the number of consecutive days where KDIGO AKI serum creatinine criteria were met over the first 7 days from the day AKI criteria were met.

We classified patients into four categories based on AKI duration: (1) resolving AKI which lasted 1–2 days (short duration AKI), (2) resolving AKI which lasted 3–7 days (medium duration AKI), (3) persistent AKI, where AKI lasted more than 7 days and (4) death during the current AKI episode. Relevant cutoffs were chosen to allow comparison with previous studies [4, 7].

### Recurrent AKI

The incidence of recurrent AKI was only examined in patients who had an initial episode of resolving AKI (e.g., short or medium duration AKI). Recurrent AKI was defined by KDIGO serum creatinine criteria using a rolling baseline. Patients were censored at hospital discharge.

### Study outcomes

Study outcomes were analyzed from the first day of AKI. Mortality was defined as death before hospital discharge up to day 30. Ventilator-free days were defined as patients being alive and breathing without ventilator assistance up to day 28. ICU-free days were defined as patients being alive and discharged from the ICU to up day 28. Patients who died before day 28 were assigned zero ventilator days and zero ICU-free days. To examine the effects of AKI duration on other organ systems, we evaluated Brussels cardiovascular failure-free days up to day 7 [11]. Patients were considered to be free from organ failure after hospital discharge. For outcome analyses, we did not include patients who died during their AKI episode, since their outcome is already well known.

### Statistical analysis

Categorical variables are presented as counts (%) and continuous variables as either median [interquartile range (IQR)] or mean ± standard deviation (SD) and compared by Chi-square test and *t* test, respectively.

We evaluated factors associated with resolving AKI versus persistent AKI using the proportional subdistribution hazards model proposed by Fine and Gray, with death as a competing risk [12]. Values were missing for a small number of subjects in the urine output or platelet count variables (*n* = 16 and *n* = 3, respectively). These were carried forward from the prior study day. PaO_2_/FiO_2_ was missing in 58 subjects on the day of AKI; 57 values were imputed using a validated nonlinear imputation of PaO_2_/FiO_2_ from SpO_2_/FiO_2_ that was developed for patients with ARDS [13]. Two patients were excluded from the multivariate analysis due to missing data in PaO_2_/FiO_2_ and vasopressor use.

In patients with an initial episode of resolving AKI (*n* = 123), we also examined the association of recurrent AKI with outcomes. Patients were stratified according to the presence or absence of recurrent AKI. Risk factors for recurrent AKI were examined using standard Cox regression and hazard ratios with 95% confidence intervals for this analysis, and the assumption of proportional hazards was tested using Schoenfeld residuals (data not shown).

A *p* value < 0.05 was considered statistically significant. All analyses were conducted using R software (http://www.R-project.org/) including the “cmprsk” R-package.

## Results

### Patients and outcomes

**Fig. 1:**
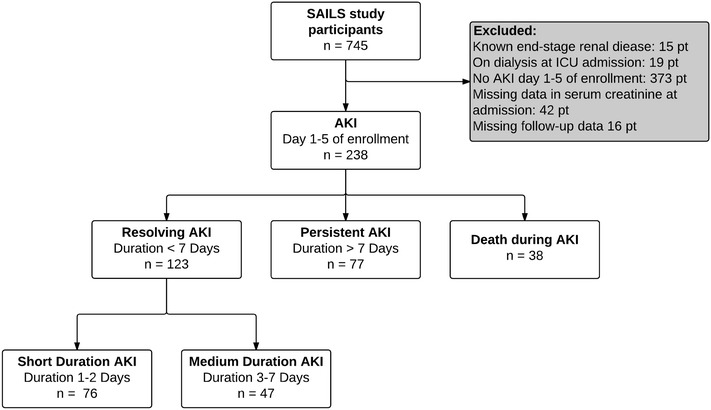
Flowchart of SAILS study participants. Patient flow in the study. Inclusion criteria in the SAILS study were development of sepsis-associated ARDS. Patients who developed AKI first 5 days of study enrollment were included in our study. Short duration AKI was defined as AKI duration of 1–2 days, medium duration AKI as 3–7 days and persistent AKI as > 7 days. *AKI* acute kidney injury, *ESRD* end-stage renal disease, *SAILS* Statins for Acutely Injured Lungs from Sepsis

Of the 745 SAILS participants, we excluded 34 patients with end-stage renal disease or who were on acute dialysis at ICU admission. Of the 711 remaining patients, 254 developed AKI between study days 1 and 5, but 16 were excluded due to critical missing data (serum creatinine or use of dialysis) to form our final cohort of 238 patients (Fig. 1).

In the overall SAILS trial, 30-day mortality was 23.9%. The patients who developed AKI were in general more severely ill, with a higher APACHE III score at enrollment, and greater use of vasopressors. In our study population (who developed AKI within the first 5 days of enrollment), 30-day mortality was 31.5%, significantly higher than those who were excluded from our analysis (*p* < 0.001). The median age was 54 (25–75% interquartile range [IQR] 40–66); 181 (76.1%) were white and 53 (22.3%) had diabetes (Table 1). The median number of days in the ICU before study enrollment was 1 (25–75% IQR 1–2), and the median day of AKI development was day 1 after study enrollment (25–75% IQR 1–3). The median baseline serum creatinine was 1.0 mg/dL (25–75% IQR 0.7–1.4), and the median serum creatinine on the day of AKI development was 1.7 mg/dL (25–75% IQR 1.2–2.6). One hundred and seventy-two (72.3%) subjects presented with KDIGO AKI stage 1, but during follow-up 56 (23.5%) progressed to either stage 2 or 3 AKI.

**Table 1:** Baseline characteristics of study population stratified by AKI duration

	SAILS patients AKI *n* = 238	Short duration AKI *n* = 76	Medium duration AKI *n* = 47	Persistent AKI *n* = 77	Death during AKI *n* = 38
*Baseline characteristics*					
Age in years	54 [40, 66]	50 [40, 65]	53 [39, 65]	54 [40, 66]	58 [47, 68]
Female gender, *n* (%)	114 (47.9)	32 (42.1)	23 (48.9)	40 (51.9)	19 (50.0)
White race, *n* (%)	181 (76.1)	58 (76.3)	36 (76.6)	58 (75.3)	29 (76.3)
Hispanic or Latino, *n* (%)	32 (13.4)	14 (18.4)	7 (14.9)	8 (10.4)	3 (7.9)
BMI (kg/m^2^)	28.6 [23.6, 34.2]	28.0 [22.5, 33.3]	26.4 [22.8, 32.1]	30.5 [25.7, 34.6]	27.9 [24.0, 34.7]
Medical admission, *n* (%)	218 (91.6)	70 (92.1)	41 (87.2)	70 (90.9)	37 (97.4)
ICU days before study enrollment *n* (%)	1 [1, 2]	1 [1, 2]	2 [1, 3]	1 [1, 2]	2 [1, 2]
Rosuvastatin therapy, *n* (%)	120 (50.4)	33 (43.4)	19 (40.4)	46 (59.7)	22 (57.9)
Day of randomization APACHE III	98 [81, 121]	92 [78, 107]	91 [73, 112]	105 [89, 123]	121 [95, 139]
*Comorbidities*					
Diabetes mellitus, *n* (%)	53 (22.3)	16 (21.1)	10 (21.3)	17 (22.1)	10 (26.3)
Hypertension, *n* (%)	112 (47.1)	35 (46.1)	20 (42.6)	35 (45.5)	22 (57.9)
Congestive heart failure, *n* (%)	16 (6.7)	2 (2.6)	5 (10.6)	8 (10.4)	1 (2.6)
Peripheral vascular disease, *n* (%)	12 (5.0)	4 (5.3)	3 (6.4)	4 (5.2)	1 (2.6)
Chronic pulmonary disease, *n* (%)	35 (14.7)	13 (17.1)	7 (14.9)	10 (13.0)	5 (13.2)
Cancer, *n* (%)	38 (16.0)	14 (18.4)	4 (8.5)	8 (10.4)	12 (31.6)
*Day of AKI development*					
Study day that AKI developed	1 [1, 3]	2 [1, 3]	1 [1, 3]	1 [1, 2]	1 [1, 2]
Urine output (mL/kg/h)	0.78 [0.28, 1.48]	1.05 [0.70, 1.81]	1.36 [0.58, 2.87]	0.43 [0.06, 0.86]	0.31 [0.12, 0.88]
Platelets × 10^6^/L	168 [74, 256]	204 [120, 292]	185 [115, 272]	143 [67, 220]	68 [26, 184]
Vasopressor use, *n* (%)	105 (44.3)	22 (28.9)	13 (27.7)	39 (51.3)	31 (81.6)
Systolic BP (mm Hg)	90 [80, 100]	94 [85, 105]	92 [82, 107]	86 [79, 98]	78 [70, 89]
PaO_2_/FiO_2_ ratio	155 [110, 220]	184 [129, 257]	192 [133, 272]	147 [106, 190]	123 [87, 151]
*AKI characteristics*					
Baseline creatinine (mg/dL)	1.0 [0.7, 1.4]	0.9 [0.7, 1.4]	0.9 [0.6, 1.3]	1.2 [0.7, 1.9]	1.0 [0.7, 1.2]
Creatinine at AKI diagnosis (mg/dL)	1.7 [1.2, 2.6]	1.4 [1.0, 2.0]	1.4 [1.1, 2.0]	2.4 [1.6, 3.8]	1.8 [1.4, 2.4]
On dialysis, *n* (%)	25 (10.5)	1 (1.3)	0 (0.0)	18 (23.4)	6 (15.8)
*KDIGO AKI stage at presentation*					
Stage 1, *n* (%)	172 (72.3)	72 (94.7)	40 (85.1)	35 (45.5)	25 (65.8)
Stage 2, *n* (%)	25 (10.5)	1 (1.3)	5 (10.6)	14 (18.2)	5 (13.2)
Stage 3, *n* (%)	41 (17.2)	3 (3.9)	2 (4.3)	28 (36.4)	8 (21.1)
*KDIGO AKI maximum severity stage day 1*–*7*					
Stage 1, *n* (%)	116 (48.7)	63 (82.9)	31 (66.0)	13 (16.9)	9 (23.7)
Stage 2, *n* (%)	36 (15.1)	5 (6.6)	12 (25.5)	11 (14.3)	8 (21.1)
Stage 3, *n* (%)	86 (36.1)	8 (10.5)	4 (8.5)	53 (68.8)	21 (55.3)

Continuous variables are presented as median and interquartile range

*AKI* acute kidney injury, *SAILS* Statins for Acutely Injured Lungs from Sepsis, *BMI* body mass index, *APACHE III* acute physiologic and chronic health evaluation III, *ICU* intensive care unit, *BP* blood pressure, *KDIGO* kidney disease: improving global outcomes, *PaO*_*2*_*/FiO*_*2*_ *ratio* partial pressure arterial oxygen/fraction of inspired oxygen

### Duration of acute kidney injury

**Fig. 2:**
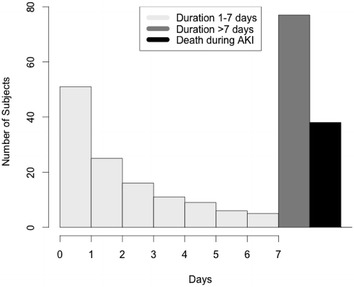
Histogram of AKI duration. Histogram of AKI duration in patients included on our study (*n* = 238) with a 7-day follow-up. One hundred and eighteen patients had an AKI duration lasting less than 7 days. Seventy-seven patients experienced AKI duration > 7 days, while 43 patients died during an AKI episode

Of the 238 study subjects, 76 (31.9%) experienced short duration AKI (1–2 days), 47 (19.7%) had medium duration AKI (3–7 days), 77 (32.4%) had persistent AKI on day 7, and 38 (16.0%) died during AKI. A histogram of patients’ AKI duration is presented in Fig. 2.

Table 1 presents baseline and AKI characteristics stratified by AKI duration. The groups with short and medium duration AKI had similar distributions of comorbidities, APACHE III score at enrollment, as well as measures of general disease severity including platelet count, use of vasopressors and PaO_2_/FiO_2_ ratio on the day of AKI development (*p* > 0.05 for listed characteristics for short duration vs. medium duration, data not shown). Patients who experienced persistent AKI or who died were sicker, with higher APACHE III at enrollment, greater use of vasopressors, lower platelet counts, and lower PaO_2_/FiO_2_ ratio on the day of AKI development. Patients with short duration or medium duration AKI presented with less severe AKI and had a lower maximum AKI stage, compared to patients with persistent AKI.

### Effect of AKI duration of outcomes

Table 2 presents outcomes stratified according to AKI duration. There were no significant differences or observable trends between the groups with short and medium duration AKI with regard to cardiovascular failure-free days, ventilator-free days, ICU-free days or 30-day mortality. Compared to patients with persistent AKI, patients with short duration AKI had more cardiovascular failure-free days (4.8 ± 2.3 vs. 3.2 ± 2.4, *p* < 0.001), ventilator-free days (17.6 ± 10.8 vs. 13.2 ± 10.0, *p* < 0.01) and ICU-free days (16.6 ± 10.7 vs. 10.8 ± 9.3, *p* < 0.01), but there was no difference in 30-day mortality (18.4 vs. 20.8%, *p* = 0.87). Further, patients with medium duration AKI had more cardiovascular failure-free days (4.8 ± 2.2 vs. 3.2 ± 2.4, *p* < 0.01), ventilator-free days (17.5 ± 10.7 vs. 13.2 ± 10.0, *p* = 0.02) and ICU-free days (16.9 ± 9.9 vs. 10.8 ± 9.3, p < 0.01) when compared to patients with persistent AKI. No difference in 30-day mortality (14.9 vs. 20.8%, *p* = 0.56) was found between patients with medium duration AKI and persistent AKI. There were no differences in duration of AKI or mortality based on treatment assignment to rosuvastatin or placebo (data not shown).

**Table 2:** Association of AKI duration with outcomes

	Short duration AKI *n* = 76	Medium duration AKI *n* = 47	Persistent AKI *n* = 77	*p* value (short vs. medium duration AKI)	*p* value (short duration vs. persistent AKI)	*p* value (medium duration vs. persistent AKI)
Cardiovascular failure-free days to day 7 (mean ± SD)	4.8 ± 2.3	4.8 ± 2.2	3.2 ± 2.4	0.98	< 0.001	0.001
Ventilator-free days to day 28 (mean ± SD)	17.6 ± 10.8	17.5 ± 10.7	13.2 ± 10.0	0.97	0.009	0.02
ICU-free days to day 28 (mean ± SD)	16.6 ± 10.7	16.9 ± 9.9	10.8 ± 9.3	0.85	0.001	0.001
Death in health care facility to day 30, *n* (%)	14 (18.4)	7 (14.9)	16 (20.8)	0.80	0.87	0.56

Cardiovascular failure was defined as the need for vasopressor or a systolic blood pressure of 90 mmHg or less. Patients who died before day 28 were assigned zero ventilator days and zero ICU-free days

*AKI* acute kidney injury, *ICU* intensive care unit, *SD* standard deviation, *NS* nonsignificant

In the sensitivity analysis where only those with maximum KDIGO AKI severity stage 1 (Additional file 1: Table 1) were analyzed, cardiovascular failure-free days, ventilator-free days, ICU-free days and 30-day mortality were virtually the same in those with short and medium duration AKI, as observed in the main analysis. A very small number of individuals had persistent AKI that remained Stage 1 (*n* = 13), so we had very limited power to detect differences in outcomes between those with persistent AKI and those with short or medium duration AKI among those with Stage 1 AKI.

### Factors associated with resolving AKI

We next examined factors associated with resolving AKI to day 7 (Table 3). In univariate analyses, higher systolic blood pressure was associated with higher rates of resolving AKI while oliguria, moderate/severe ARDS and higher serum creatinine on the day of AKI were associated with lower rates of resolving AKI and remained significant predictors in multivariate analysis.

**Table 3:** Factors associated with resolving AKI

*n* = 236	Univariate Cox regression (hazard ratio)		*p* value	Multivariate Cox regression (hazard ratio)		*p* value
	HR	(95% CI)		HR	(95% CI)	
Age, per 10 year increase	0.94	0.86–1.03	0.19			
Female gender	0.85	0.61–1.17	0.31			
White race	1.03	0.70–1.51	0.88			
Hispanic or Latino ethnic group	1.50	0.98–2.30	0.06			
BMI	1.00	0.98–1.02	0.88			
Diabetes mellitus	0.91	0.61–1.34	0.63			
History of hypertension	0.91	0.65–1.26	0.55			
Platelet count			< 0.001			0.27
> 150 × 10^9^/L	1			1		
< 150 × 10^9^/L	0.54	0.38–0.78		0.80	0.53–1.19	
Urine output			< 0.0001			0.01
> 0.5 mL/kg/h	1			1		
< 0.5 mL/kg/h	0.31	0.20–0.47		0.53	0.32–0.87	
PaO_2_/FiO_2_ ratio			< 0.0001			0.001
> 200	1			1		
< 200	0.52	0.38–0.71		0.59	0.44–0.81	
Creatinine mg/dl, per unit increase	0.59	0.48–0.74	< 0.0001	0.71	0.57–0.89	0.003
Systolic BP, per 10 mm Hg increase	1.15	1.05–1.25	0.002	1.09	0.99–1.20	0.07
Vasopressor use	0.46	0.32–0.67	< 0.0001	0.91	0.59–1.41	0.68

Factors associated with resolving AKI, defined as an AKI duration of less than 7 days, analyzed by the proportional subdistribution hazards model proposed by Fine and Gray, with death as a competing risk. Two patients were excluded from the original population (*n* = 238) due to missing values

*AKI* acute kidney injury, *BMI* body mass index, *BP* blood pressure, *PaO*_*2*_*/FiO*_*2*_ *ratio* partial pressure arterial oxygen/fraction of inspired oxygen

### Impact of recurrent AKI on outcomes and risk factors for recurrent AKI

We examined the incidence of recurrent AKI in those with an initial episode of resolving AKI. Of the 123 subjects, 43 subjects (35.0%) met criteria for AKI again during in the hospital admission. When outcomes were examined in patients with or without recurrent AKI (Table 4), there were still no differences or trends in outcomes between those short versus medium duration AKI, similar to our main analysis. However, those with an initial episode of short and medium duration AKI *without* recurrent AKI had better outcomes than those who experienced an episode of recurrent AKI, with more ventilator-free days (mean ± SD, 20.2 ± 9.8 vs. 12.7 ± 10.6, *p* = 0.003), ICU-free days (mean ± SD, 19.5 ± 9.1 vs. 11.4 ± 10.7, *p* = 0.001), and lower 30-day mortality (11.2 vs. 27.9%, *p* = 0.039). Similar results were observed when the analysis was restricted to patients with a maximum of stage 1 AKI and stratified according to the presence or absence of recurrent AKI (data not shown). However, no baseline factors were found to be significantly associated with development of recurrent AKI in the univariate Cox regression analysis (Additional file 2: Table 2).

**Table 4:** Association of recurrent AKI with outcomes

*n* = 123	No recurrent AKI		*p* value*	Recurrent AKI	*p* value^§^
	Short duration AKI *n* = 46	Medium duration AKI *n* = 34		Short and medium duration AKI *n* = 43	
Cardiovascular failure-free days to day 7 (mean ± SD)	5.1 ± 2.3	4.8 ± 2.3	0.47	4.4 ± 2.3	0.06
Ventilator-free days to day 28 (mean ± SD)	20.4 ± 10.0	20.0 ± 9.8	0.89	12.7 ± 10.6	0.003
ICU-free days to day 28 (mean ± SD)	19.7 ± 9.5	19.4 ± 8.7	0.89	11.4 ± 10.7	0.001
Death in health care facility to day 30, *n* (%)	5 (10.9)	4 (11.8)	1.00	12 (27.9)	0.04

Cardiovascular failure was defined as the need for vasopressor or a systolic blood pressure of 90 mmHg or less. Patients who died before day 28 were assigned zero ventilator days and zero ICU-free days

*AKI* acute kidney injury, *ICU* intensive care unit, *SD* standard deviation, *NS* nonsignificant

*Short duration AKI without recurrent AKI vs. medium duration AKI without recurrent AKI

^§^Short and medium duration AKI without recurrent AKI versus short and medium duration AKI with recurrent AKI

## Discussion

AKI remains a disease with poor outcomes for which there are no treatments, apart from supportive care. Duration of AKI has been recognized as an important factor associated with outcomes following AKI. It has been implied that differences in AKI duration may represent distinct phenotypes of AKI due to etiology of injury, but these views are still to be confirmed at a mechanistic level [6, 8, 14]. In this study, we explored the effect of AKI duration on outcomes in a cohort of critically ill patients with sepsis-associated ARDS.

We found that 31.9% of the patients had an AKI duration of less than 48 h, demonstrating that short duration AKI is very common. Consequently, to further examine the effect of short duration AKI on outcomes, we divided those with resolving AKI into two subgroups of short and medium duration AKI. Although we expected the patients with short AKI to have lower severity of illness than patients with medium duration AKI, there were no differences between the groups with regard to comorbidities, AKI severity or general disease severity measurements. Patients with short duration AKI had similar outcomes compared to the patients with medium duration AKI, and these results were confirmed in two subsequent analyses. First, to examine whether the results were due to differences in AKI severity, we restricted our analysis to those whose maximum AKI severity was Stage 1. Second, we hypothesized that those with short duration AKI might have an increased risk of adverse outcomes due to recurrent AKI episodes and therefore stratified our analysis by the presence or absence of recurrent AKI. Again, there were no differences in outcomes, suggesting that short duration AKI is not associated with better outcomes than medium duration AKI in critically ill patients.

A significant proportion of patients (32.4%) had persistent kidney failure lasting 7 or more days, and these patients experienced poorer clinical outcomes including increased length of ICU stay, time on the ventilator and days with cardiovascular failure. This emphasizes the importance of identifying high-risk patients who may benefit the most from early interventions and novel treatments. Along the same lines, those with quick and spontaneous resolving AKI may dilute trials and perhaps should not be the target of new treatment trials. Here, we found that lack of oliguria, lower serum creatinine on the day of AKI and having mild ARDS were associated with increased likelihood of resolving AKI.

Patients with recurrent AKI were found to have worse short-term outcomes. It has previously been reported that recurrent AKI episodes are associated with an increased risk of CKD progression and other adverse long-term outcomes [14], but, to the best of our knowledge, this is one of the first studies to examine the effect of recurrent AKI in critically ill patients and during a single hospitalization. We went on to examine risk factors for recurrent AKI and were not able to identify any, other than a trend for gender and ethnicity. This may reflect the fact that we only studied risk factors at baseline and upon development of the first AKI episode, whereas future or time-updated risk factors (e.g., new or recurrent sepsis, repeated exposures to nephrotoxins) are likely the strongest drivers of recurrent AKI. Alternatively, this may have been due to a lack of power given our relatively small sample size or the exclusion of certain very high-risk populations (e.g., those with advanced heart failure or liver failure) from the overall population. A similar study examined predictors of recurrent AKI after renal transplantation [15] and was unable to identify any risk factors, while other studies have mostly focused on predictors of a second hospitalization with AKI, rather than a second episode during a single hospitalization [16].

Our overall results differ somewhat from other studies, which have highlighted the importance of AKI duration and especially transient AKI. Many of these studies have focused on less severely ill patients or have only included patients who survive hospitalization [17]. In contrast, our population of critically ill patients had multi-organ failure with sepsis and ARDS in addition to AKI, and the in-hospital mortality rate of this patient group was very high. While other studies have only focused on mortality as primary endpoint, we here report the influence of AKI duration on other relevant outcomes along with mortality while accounting for AKI severity and recurrent AKI status.

Potential limitations of our study include the very specific nature of our population, all of whom were critically ill adults with sepsis-associated ARDS who developed AKI. However, this is an extremely well-characterized population from a large randomized clinical trial, where a number of aspects of care including mechanical ventilation and fluid management were protocolized, resulting in less concern about variations in clinical care and the effect of these variations on outcomes. Furthermore, there has been much interest in the interplay of ARDS and AKI, which thought to be closely connected as part of multi-organ dysfunction, with the presence of both diseases associated with worse clinical outcomes [18]. Although more studies are needed to determine the direct effects of ARDS on AKI, our study focuses on a well-recognized and important clinical population. Outpatient creatinine measurements were unfortunately not unavailable, but this is often the case the clinical settings, where many patients lack an outpatient baseline creatinine. As part of the study protocol, serum creatinine values were recorded for up to 48 h before study enrollment, which allowed us to use these serum creatinine values as baseline values and to calculate a rolling 48-h window for AKI ascertainment, rather than using an imputed baseline or nadir serum creatinine as baseline. Our study is greatly strengthened by its richness and completeness in clinical variables obtained as part of a multicenter clinical trial as well as the racial/ethnic diversity of the study population.

## Conclusions

In summary, we evaluated the association of AKI duration on outcomes in a cohort of critically ill patients with sepsis-associated ARDS. We found no differences in outcomes in patients with short duration AKI compared to those with medium duration AKI, while persistent AKI was associated with worse outcomes. Compared to patients with persistent AKI or who died during AKI, patients with resolving AKI were less likely to have oliguria, moderate/severe ARDS or higher serum creatinine on the day of AKI. We found that recurrent AKI was associated with poorer clinical outcomes, but no clinical factors at baseline could predict the development of recurrent AKI. Our findings suggest that the ideal population for AKI clinical trials in patients with ARDS may be those with persistent AKI or at increased risk of persistent AKI, since the impact of short and medium duration AKI on clinical outcomes is modest.

## Additional files

**Additional file 1.** Association of stage 1 AKI with outcomes stratified by AKI duration. Patients with a maximum of KDIGO AKI stage 1 during 7 days of follow-up.

**Additional file 2.** Risk factors for development of recurrent AKI in patients with an initial episode of resolving AKI, analysed using Cox regression.

## Abbreviations

AKI: acute kidney injury
ARDS: acute respiratory distress syndrome
CKD: chronic kidney disease
ICU: intensive care unit
IQR: interquartile range
KDIGO: kidney disease: improving global outcomes (KDIGO), guidelines form 2012
