# Supplementary material for: Duration of acute kidney injury in critically ill patients
Source: Ann Intensive Care. 2018 Feb 23;8:30. doi: 10.1186/s13613-018-0374-x (PMC5823799; doi:10.1186/s13613-018-0374-x)
Supplement: Supplementary file 1 — Additional file 1. Association of stage 1 AKI with outcomes stratified by AKI duration. Patients with a maximum of KDIGO AKI stage 1 during 7 days of follow-up. [file 13613_2018_374_MOESM1_ESM.docx]

**ADDITIONAL FILE 1:**

**Additional Table 1: Association of Stage 1 AKI Stratified by AKI duration with Outcomes**

| KDIGO AKI Stage 1, n = 107 | Short Duration AKI  n = 63 | Medium Duration AKI  n = 31 | Persistent AKI  n = 13 | P-value* |
| --- | --- | --- | --- | --- |
| Cardiovascular failure free days to day 7 (mean ± SD) | 4.8 ± 2.2 | 4.9 ± 2.0 | 3.7 ± 2.1 | 0.20 |
| Ventilator free days to day 28 (mean ± SD) | 18.0 ± 11.1 | 16.7 ± 11.7 | 12.7 ± 10.0 | 0.30 |
| ICU free days to day 28 (mean ± SD) | 17.1 ± 10.9 | 16.9 ± 10.6 | 10.4 ± 9.0 | 0.11 |
| Death in health care facility to day 30 n (%) | 12 (19.0) | 6 (19.4) | 4 (30.8) | 0.62 |

Patients with a maximum of KDIGO AKI stage 1 during 7 days of follow-up.

*ANOVA and Chi2-test performed between all three groups.

Abbreviations: AKI: Acute kidney injury, ICU: Intensive care unit, SD: Standard deviation, NS: Non-significant.

Cardiovascular failure was defined as the need for vasopressor or a systolic blood pressure of 90 mmHg or less. Patients who died before day 28 were assigned zero ventilator days and zero ICU-free days.
