# Supplementary material for: Duration of acute kidney injury in critically ill patients
Source: Ann Intensive Care. 2018 Feb 23;8:30. doi: 10.1186/s13613-018-0374-x (PMC5823799; doi:10.1186/s13613-018-0374-x)
Supplement: Supplementary file 2 — Additional file 2. Risk factors for development of recurrent AKI in patients with an initial episode of resolving AKI, analysed using Cox regression. [file 13613_2018_374_MOESM2_ESM.docx]

**ADDITIONAL FILE 2:**

**Additional Table 2: Risk factors for Development of Recurrent AKI**

| n = 123 | Univariate  Cox regression | |  |
| --- | --- | --- | --- |
|  | HR | (95%-CI) | P Value |
| Age, per 10 year increase | 1.15 | 0.74–1.80 | 0.54 |
| Female gender | 0.56 | 0.30–1.05 | 0.07 |
| White race | 0.65 | 0.34–1.25 | 0.19 |
| Hispanic or Latino ethnic group | 1.92 | 0.94–3.91 | 0.07 |
| BMI | 1.12 | 0.85–1.49 | 0.41 |
| Diabetes Mellitus | 1.56 | 0.80–3.05 | 0.19 |
| History of hypertension | 0.75 | 0.41–1.39 | 0.36 |
| Platelet count |  |  | 0.90 |
| > 150 x 10^9^/L | 1 |  |  |
| < 150 x 10^9^/L | 0.96 | 0.52–1.78 |  |
| Urine output |  |  | 0.52 |
| >0.5 mL/kg/h | 1 |  |  |
| <0.5 mL/kg/h | 1.25 | 0.63–2.49 |  |
| PaO_2_/FiO_2_ ratio |  |  | 0.77 |
| >200 | 1 |  |  |
| <200 | 1.10 | 0.59–2.03 |  |
| Creatinine mg/dl, per unit increase | 0.84 | 0.54–1.32 | 0.45 |
| Systolic BP, per 10 mm Hg increase | 0.98 | 0.70–1.38 | 0.91 |
| Vasopressor use | 1.08 | 0.57–2.02 | 0.81 |

Risk factors for Development of Recurrent AKI in patients with an initial episode of resolving AKI, analysed using Cox regression.

Abbreviations: AKI: Acute kidney injury, BMI: Body mass index, BP: Blood pressure, PaO2/FiO2 ratio: Partial pressure arterial oxygen / Fraction of inspired oxygen.
